# Immunogenicity of two‐dose sinopharm BBIB‐CorV vaccine in Morocco: One‐year follow‐up and neutralizing activity against severe acute respiratory syndrome coronavirus 2 variants of concern

**DOI:** 10.1002/iid3.1359

**Published:** 2024-11-12

**Authors:** Maha Ouarab, Elarbi Bouaiti, Zineb Rhazzar, Hicham El Annaz, Safae el kochri, Mouhssine Hemlali, Hamza Ghammaz, Omar Nyabi, Karim el Bakkouri, Nadia Touil, Mostafa Elouennass, Lamiae Belayachi, Jean Luc Gala, Khalid Ennibi, Elmostafa El Fahime

**Affiliations:** ^1^ Neuroscience and Neurogenetics Research Team, Faculty of Medicine and Pharmacy University Mohammed V of Rabat Rabat Morocco; ^2^ Molecular Biology and Functional Genomics Platform National Center for Scientific and Technical Research (CNRST) Rabat Morocco; ^3^ Center of Virology, Infectious and Tropical Diseases Mohammed V Military Teaching Hospital Rabat Morocco; ^4^ Cell Culture Unit, Center of Virology, Infectious and Tropical Diseases Mohammed V Military Teaching Hospital Rabat Morocco; ^5^ Immunopathology Research Team (ERIP), Faculty of Medicine and Pharmacy University Mohammed V Rabat Morocco; ^6^ Faculty of Medicine and Pharmacy University Hassan II Casablanca Morocco; ^7^ Center for Applied Molecular Technologies (CTMA), Institute of Clinical and Experimental Research Université Catholique de Louvain Brussels Belgium; ^8^ Genomic Center for Human Pathologies (GENOPATH), Faculty of Medicine and Pharmacy University Mohammed V Rabat Morocco; ^9^ Department of Bacteriology Mohammed V Military Teaching Hospital Rabat Morocco; ^10^ Health Sciences Research Center, BioMed Unit University International of Rabat (UIR) Sala‐Al Jadida Morocco; ^11^ Mohammed VI Center of Research and Innovation Mohammed VI Univeristy of Sciences and Health Rabat Morocco

**Keywords:** BBIBP‐CorV Sinopharm, immunogenicity, microneutralization assay, neutralizing antibodies, SARS‐CoV‐2

## Abstract

**Background:**

This study aimed to evaluate the immunogenicity of a two‐dose Sinopharm BBIB‐CorV (Vero cells) vaccine against SARS‐CoV‐2, at 28 days, 6 months, and 1‐year postvaccination. And assess the capacity of two‐dose vaccine recipients to neutralize SARS‐CoV‐2 strains B.1 (Wuhan/D614G), B.1.1.7 (Alpha), AY.33 (Delta), or BA.5.2.2 (Omicron) variants of concern (VOCs).

**Methods:**

A prospective matched case–control cohort study was conducted at the Military Hospital of Rabat, Morocco between February 2021 and 2022. Immunogenicity was evaluated by standard Microneutralization (MN) assay against four variants (Wuhan D614G, Alpha, Delta, and Omicron).

**Results:**

The overall positive neutralizing rates for vaccine recipients against B.1 D614G were 72.09%, 74.82%, and 75.19% on 28‐, 180‐, 365‐ day respectively. The proportion of NAbs targeting the Wuhan D614G, and Alpha variants under the BBIBP‐CorV vaccination was high on Day 28‐ and 6 months postvaccination.

**Conclusion:**

The immunogenic response to the newly emerging SARS‐CoV‐2 variants of concern (VOCs), such as Delta and Omicron was comparatively reduced. As a result, it is recommended that additional boost vaccinations be considered.

## Graphical abstract

This study assessed the immune response to the Sinopharm BBIB‐CorV vaccine against SARS‐CoV‐2 over a period of 1 year, using a two‐dose regimen. Conducted at the Military Hospital of Rabat, Morocco, the study found that additional booster vaccinations may be necessary based on the observed immunogenicity against various virus variants.

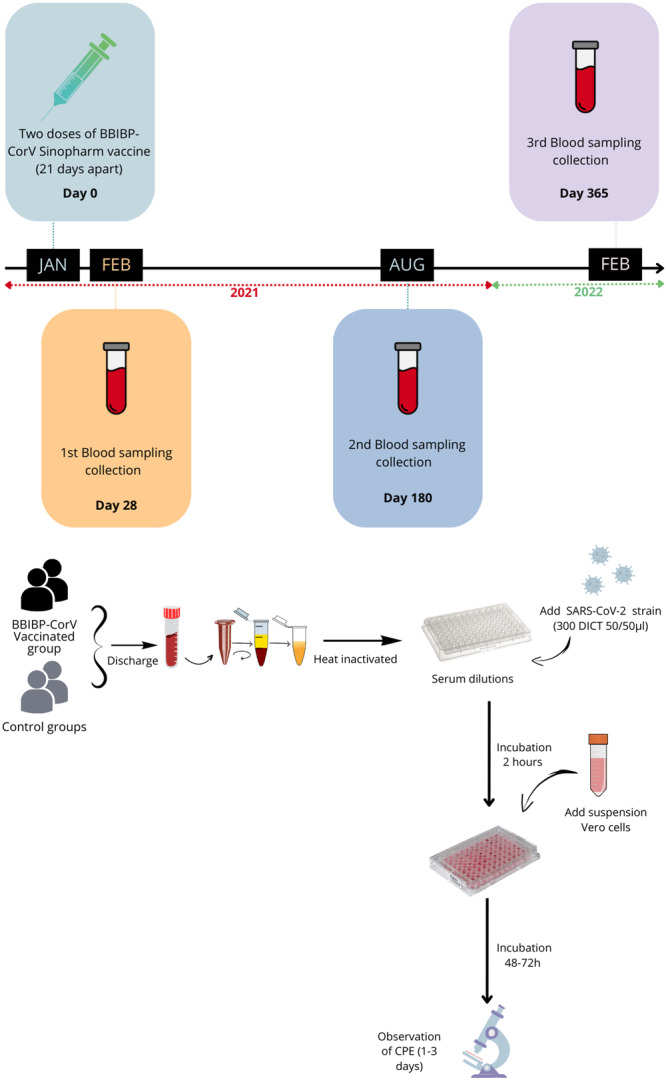

## 1 INTRODUCTION

The development of safe and effective vaccines against Coronavirus disease 2019 (COVID‐19) constituted a pivotal milestone that facilitated the restarting of normal societal activities. Various vaccine platforms have been employed in response to the pandemic caused by the severe acute respiratory syndrome coronavirus 2 (SARS‐CoV‐2) including inactivated vaccines, mRNA vaccines, adenoviral‐based vector vaccines, recombinant protein subunit, and the recently bivalent mRNA vaccines.^1^ All vaccines showed minor side effects and exhibited high immunogenicity against SARS‐CoV‐2 during the first weeks following the second dose.^2^ In Morocco, the vaccination campaign for health workers, uniformed service members, and civilian employees started in late January 2021, following the guidelines set forth by the National Scientific and Technical Committee.^3^ By April 15, 2021, the military population had received the complete vaccination regimen (two doses) of either BBIBP‐CorV (Sinopharm), Pfizer‐BioNTech's BNT162b2, or the Oxford/AstraZeneca COVID‐19 vaccine (ChAdOx1 nCoV‐19), all of which were authorized for emergency use by Morocco's health authorities in August 2021.^4^ Over the course of the previous 24 months, Morocco has participated in a large multicountry Phase 3 trial evaluating the efficacy of a two‐dose regimen of the BBIBP‐CorV (Sinopharm) vaccine The data about the vaccine's effectiveness (VE) is currently being under investigation. The real‐world country conditions yielded an 89% VE for primary series vaccinations using BBIBP‐CorV against hospitalization due to serious or critical COVID‐19^5^ BBIBP‐CorV. VE against symptomatic wild‐type COVID‐19 was 78.1% during the phase III clinical trial in the United Arab Emirates^6^ and was 84% against COVID‐19 related mortality among those aged ≥60 years in Argentina where Alpha, Gamma, and Lambda were the predominant circulating SARS‐CoV‐2 VOCs.^7^ The efficacy of the two‐dose vaccine was assessed in Hungary and was found to be comparatively lower (69%) in the presence of the Alpha variant, which was the prevailing variant at the time.^8^

Although essential in evaluating vaccine immunogenicity (VI), the aforementioned studies did not analyze the performance of VI against SARS‐CoV‐2 variants present under real‐world conditions especially in light of the predominance of Omicron variants in last year. The ability of the two‐dose BBIBP‐CorV inactivated vaccine to protect against Whuhan‐Hu‐1, Alpha, Beta, Gamma, Iota, and Delta variants was established recently by the World Health Organization (WHO).^9^ Health workers (*n* = 760) exhibited a high rate of seroconversion (91.84%) and had neutralizing antibodies (NAbs) against the spike protein (S) from the wild‐type strain (Wuhan‐Hu‐1) on Day 28 after the second dose. The study found that even after 180 days, the rate of positive cases with specific neutralizing antibodies (NAbs) could still be detected in 86.06% of the samples. However, only 34.68% of the 470 vaccine‐elicited sera had NAbs against the variants of concern (VOCs) that were included in the study.

The current study aims to evaluate and compare the immunogenicity of the two‐dose BBIBP‐CorV (Sinopharm) inactivated vaccine, administered according to the same vaccine schedules as previously studied by the WHO,^9^ against various strains of concern (VOCs), including B.1 (Wuhan/D614G), B.1.1.7 (Alpha), AY.33 (Delta), and BA.5.2.20 (Omicron) variants. The standard Microneutralization (MN) assay was utilized for this purpose. Furthermore, we incorporated sera obtained from control cohorts to assess and oversee the transmission of COVID‐19. Serological assays that measure the antibody responses against SARS‐CoV‐2 infection provide a cost‐effective means of detection that can complement the existing surveillance tools. Furthermore, the data of COVID‐19‐positive cases within our medical facility during the time frame (28‐, 180‐, and 365 days postvaccination) is provided to conduct comparative analyses.

## 2 MATERIALS AND METHODS

### 2.1 Data collection of Covid‐19 positive cases

The COVID‐19 testing data for the study was collected from the electronic database of the Military Hospital of Rabat, covering the period March 2020 and January 2023, from individuals tested for the virus, regardless of symptoms. The institutional electronic database is a component of the national surveillance data sets. The testing for SARS‐CoV‐2 adheres to the strategy recommended by the Ministry of Health, with diagnosis relying on real‐time polymerase chain reaction (RT‐PCR) techniques.

### 2.2 Study design and participants

A prospective matched case–control study was conducted for 1 year (February 2021–2022) among health participants who were selected from the Military Hospital of Rabat and participated in a study involving the administration of two doses of inactivated SARS‐CoV‐2 vaccine BBIBP‐CorV, Sinopharm developed by Beijing Institute of Biological Products.^10^

The control group was collected at the same time scale and studied with the same methods as the vaccinated participants. However, during the 1‐year follow‐up, we had some missing data due to many sources of bias. Some participants were unreachable, some results were Invalid. In addition, we ran out of some participants' sera for the analysis against all four SARS‐CoV‐2 VOCs used in our study.

### 2.3 Inclusion and exclusion criteria

We included healthy healthcare professionals (*n* = 129) affiliated with the military hospital in Rabat, included not having serum‐specific antibodies against the B.1 D614G variant during screening, testing negative for SARS‐CoV‐2 virus nucleic acid using the GeneFinder assay through RT‐PCR, and expressing a willingness to undergo monitoring for 1 year. Additionally, they were willing to undergo testing for NAbs neutralizing antibody activity against any potential variants that may emerge within 1 year of the follow‐up.

All participants were requested to report any adverse reactions to the vaccine as well as any instances of COVID‐19 infection or contact with a confirmed case during the follow‐up study. The serological analysis included samples taken at the onset of the investigation, as well as at 6‐month and 1‐year intervals thereafter.

**Figure 1:**
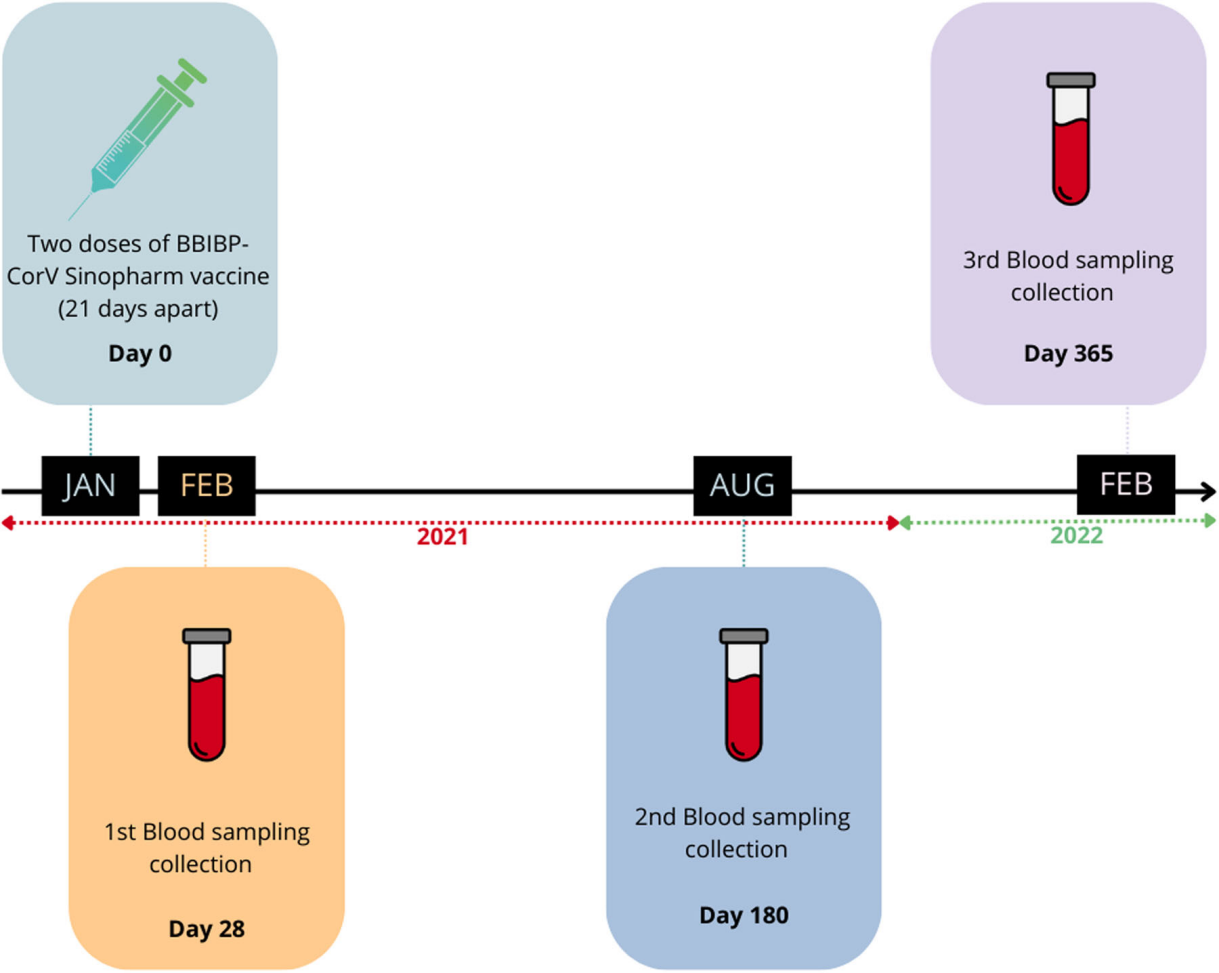
Timeline study of the matched case–control cohort.

Figure 1 shows an overview of our study with the key time scale of sera collection (28‐, 180‐, and 365‐day two‐dose Sinopharm postvaccination).

### 2.4 Microneutralization assay (MN)

#### 2.4.1 Cells

The cell culture reagents were procured from Gibco. The Vero‐E6 and Vero cells (derived from the kidney of the African green monkey) were kindly provided by Dr Ducatez from INRA, Toulouse, France, and Biopharma, Morocco. Pr. J.L Gala from UCL, Brussels, Belgium provided the *Homo sapiens* Embryonic Kidney Epithelial stable Cells (HEK‐293T) Expressing Transmembrane Protease, Serine 2 (TMPRSS2), and Human Angiotensin‐Converting Enzyme 2 (ACE2) (ATCC® NR‐55293_12‐2021 [293TACE2.TMPRSS2 (mCherry)] or 293T‐ACE2.TMPRSS2 (mCherry) stable cell lines. The Cells were cultured in a growth medium consisting of Dulbecco's Modified Eagle's Medium containing (DMEM) containing 4 mM L‐glutamine, 4500 mg per L glucose, 1 mM sodium pyruvate, 100 U/mL of penicillin‐streptomycin (1x Pen‐Strep) and 1500 mg per L sodium bicarbonate. The medium was further supplemented with 10% heat‐inactivated fetal bovine serum. The Cells were subjected to culture conditions maintained at a temperature of 37°C and a 5% CO_2_.

#### 2.4.2 Virus strains

The identification and characterization of SARS‐CoV‐2 variants from individuals who tested positive and were hospitalized during various waves of the COVID‐19 pandemic were conducted as part of the COVID‐19 surveillance initiative, which was funded by the Hassan II Academy of Science and Technology and the Ministry of Higher Education, Scientific Research, and Innovation in Morocco. The nasopharyngeal samples were subjected to diagnostic and sequencing procedures as outlined in references^11^ and Rhazzar and colleagues (in preparation, 2024). These procedures were conducted with the approval of the Biomedical Research Ethics Committee of Med V University's Faculty of Medicine and Pharmacy in Rabat, Morocco, under the auspices of approval number (17/20). A total of four samples of SARS‐CoV‐2 were utilized. The samples analyzed in this study consisted of four distinct variants, namely B.1CA8 (collected in October 2020), B.1.1.7CA3 (collected in March 2022), AY.33CA3, and BA.5.2.20CA3 (Table 1), representing the B.1, Alpha, Delta, and Omicron lineages, respectively. The sequences pertaining to the strains can be accessed through the Global Initiative on Sharing All Influenza Data (GISAID) and are identified by the accession numbers EPI_ISL_17167250, EPI_ISL_17155879, EPI_ISL_17167251, and EPI_ISL_17179367, which can be found at https://www.gisaid.org/.

**Table 1:** The AA changes in the S protein of the four SARS‐CoV‐2 strains (B.1, B.1.1.7, AY.33, and BA5.2.20) used for micro‐neutralizing assay.

Accession number	Clade	Lineage	Cell passage numbers	AA mutations in Spike Protein
EPI_ISL_17167250	20A	B.1	P3 vero cells	R78M, D614G
EPI_ISL_17155879	20I (Alpha)	B.1.1.7	P3 vero cells	H69‐, V70‐, N501Y, A570D, D614G, R681H, T716I, S982A, D1118H
EPI_ISL_17167251	21J (Delta)	AY.33	P3 vero cells	E156‐, F157‐, T19R, T29A, R158G, T250I, L452R, Q613H, D614G, P681R
EPI_ISL_17179367	22B (Omicron)	BA.5.2.20	P3 vero cells	T19I, L24‐, P25‐, P26‐, H69‐,V70‐, A27S, G142D, V213G, G339D, S371F, S373P, S375F, T376A, D405N, R408S, K417N, N440K, L452R, S477N, T478K, E484A, F486V, D614G, H655Y, N679K, P681H, N764K, D796Y, Q954H, N969K

*Note*: This table also indicates the lineages and GISAID accession number of these strains.

The “Centre de Virologie & Maladies Infectieuses Tropicales” (CVMIT) conducted the isolation and propagation of live pathogenic SARS‐CoV‐2 viruses in a BSL‐3 containment, adhering to the guidelines set forth by the WHO. The isolation of viruses from clinical samples was conducted following the protocol outlined by the CDC, as detailed in reference.^12^ In summary, the samples underwent filtration using a 0.2 µm filter and were subsequently diluted in DMEM containing 2% FBS and 1x Pen‐Strep. The monolayers were examined daily using a light microscope to observe cytopathic effect (CPE) emergence up to 96 h after infection (hpi). Except for the Omicron variant, which was isolated on 293TACE2.TMPRSS2, all the other strains were effectively isolated on Vero‐E6. Following the second passage, various strains of SARS‐CoV‐2 were cultured on Vero cells and were inoculated with a viral multiplicity of infection (MOI) of 0.0001.

To evaluate the viral genetic alterations caused by the process of culturing, we conducted a comprehensive analysis of all viral genomic mutations present in individual Vero cells using whole viral genome sequencing, which was carried out in a manner consistent with the methodology employed for the original clinical samples.

#### 2.4.3 Sera panel used for micro‐neutralization

##### Control sera panel for micro‐neutralization assay validation

For each run, this study utilized a group of convalescent serum obtained from five patients who had previously recovered from SARS‐CoV‐2 infection, with varying levels of neutralizing antibodies (with an average titer of 64). Additionally, two prepandemic sera samples from our Biobank were used as interassay controls. The objective was to evaluate the virus neutralization capability against a prevalent worldwide SARS‐CoV‐2 isolate carrying the D614G mutation (identified as EPI_ISL_17167250).

##### Micro‐neutralization test (MNT)

**Figure 2:**
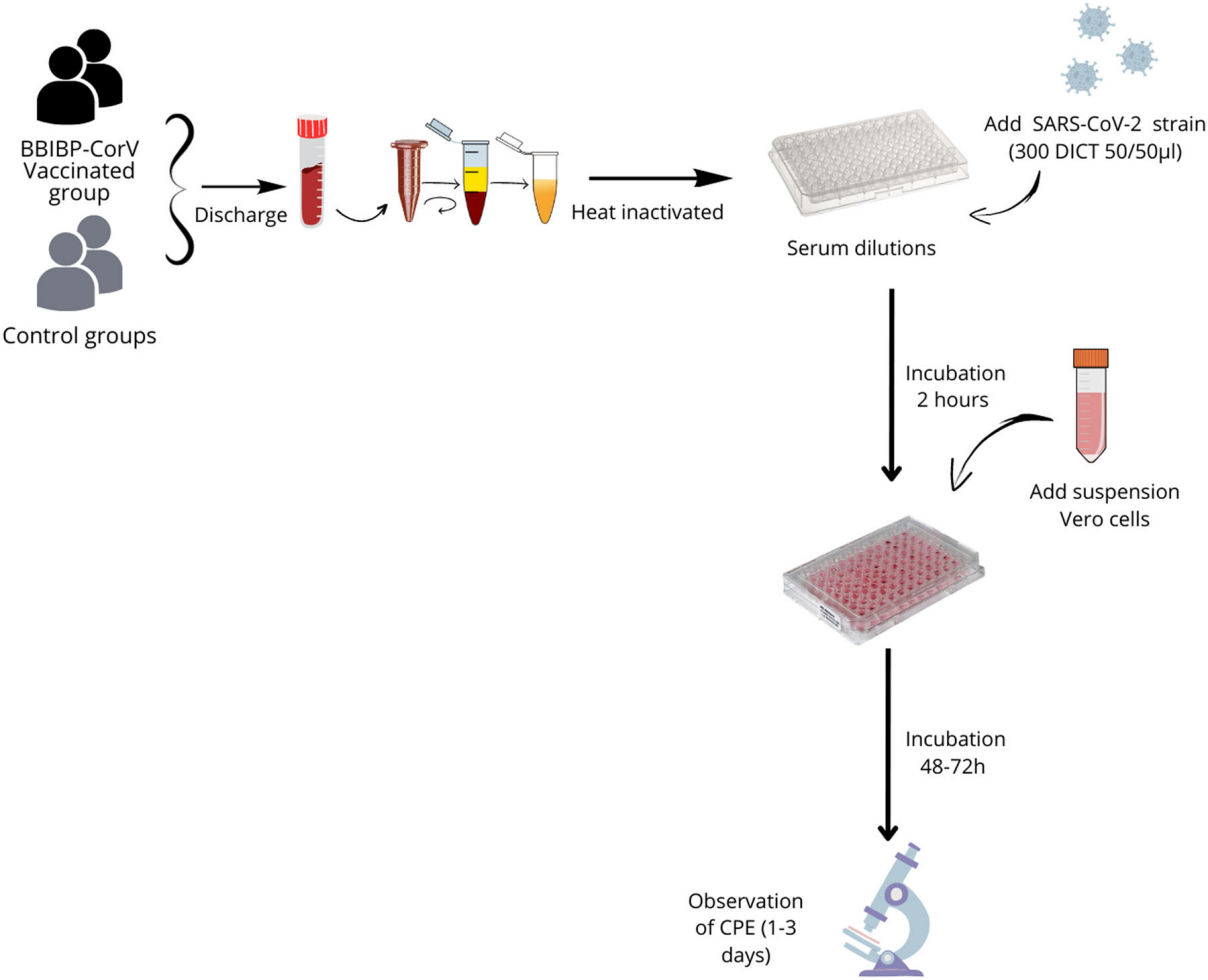
The standard micro‐neutralization test (MNT) platform based on the live virus on Vero cells performed using the four isolated SARS‐CoV‐2 variants.

The standard MNT was conducted using a live virus platform^13^ as previously described by Touil et al.^14^ with some modifications (Figure 2). Briefly, in this experiment, heat‐inactivated sera underwent four‐fold serial dilutions using DMEM. The dilutions were performed in 96‐well cell culture plates with a total volume of 50 µL. Subsequently, 50 µL of a viral suspension containing 300 TCID50 (tissue culture infectious dose 50) was added into every well, followed by a 2 h incubation period. After this, 3.10^5^ Vero cells were added to each well, and the mixture was maintained at 37°C with 5% CO_2_ for an additional 48 h. The wells were scrutinized to detect the cytopathic effect (CPE), and the titers of neutralizing antibodies were calculated as the log_10_ of the reciprocal dilution of antibody required to neutralize 50% of 300 TCID50 of the virus.

To minimize interassay variation, the plate consistently incorporated sera from identical vaccines collected at various time points. The virus back titration was conducted with negative and convalescent sera for each run. A neutralizing antibody potency that is less than 1:4 was deemed as a negative outcome. NT50 values of 1:4‐1:8 or ≥16 were classified as moderate and high, respectively, based on the NT50 values obtained from convalescent controls.

### 2.5 Statistical analysis

The analysis of quantitative data was conducted using Epi info version 7. Measures such as medians, and interquartile range proportions were computed. Meanwhile, qualitative data was analyzed and represented by frequencies and percentages. The *χ* ^2^ statistical test was used to compare the two‐dose BBIP‐CorV vaccinated with the control groups at identical time intervals.

The Wilcoxon nonparametric sign‐and‐rank statistical test was utilized to compare the NAbs within the control group. A *p* < 0.05 is defined as a statistically significant result.

In this study, we observed distinctive patterns among individuals categorized as COVID‐19 positive based on RT‐PCR tests, with *C*_t_ values less than 30. Among this subset, the presence of neutralizing activity measured by the CPE, was a significant determinant. Specifically, individuals exhibiting a titer of neutralizing antibodies exceeding 4 were considered positive.

We evaluated a cohort of participants who received two doses BBIBP‐CorV Sinopharm vaccine (21 days apart). Those are considered vaccine recipients, while the other group of individuals is deemed unvaccinated.

These outcomes shed light on the heterogeneity of the immunogenicity of a two‐dose regimen. Variability was observed in the immune response elicited by the two‐dose BBIBP‐CorV Sinopharm vaccine, with some individuals displaying robust immune responses, while others exhibited more subdued reactions.

To evaluate whether the neutralizing activity is due to the BBIBP‐CorV Sinopharm two‐dose vaccination or to field COVID‐19 infection. We ran a complementary statistical analysis of the attributable fraction (AF). This factor is defined as the proportion of incidents in the population attributable to the risk factor. It is calculated using the formula: $\mathrm{AF}=\frac{\mathrm{OR}-1}{\mathrm{OR}}$ (with OR = odds ratio).

$$\mathrm{AF}=\frac{\mathrm{OR}-1}{\mathrm{OR}}$$

L'odds ratio is a factor that measures the probabilities of an event happening in one group compared to the probabilities of the same event happening in another group. It is a statistic that quantifies the strength of the association between two groups, A and B. It is calculated using the formula: $\mathrm{OR}=\frac{b/d}{a/c}=\;\frac{a.d}{b.c}$, with:

a) The number of vaccinated participants with a positive neutralizing activity.
b) The number of vaccinated participants with a negative neutralizing activity.
c) The number of controls with a positive neutralizing activity.
d) The number of controls with a negative neutralizing activity.

### 2.6 Ethical considerations

The study protocol is under the number CEFCZ/AB/30/01/2021 folder “PR_RFG_2021.” The informed consent process was obtained from study participants before the study conduct. The participant's identities were anonymized with restricted access to the authors.

## 3 RESULTS

### 3.1 COVID‐19 positive cases in our CVMIT cohort between March 2020 and January 2023

**Figure 3:**
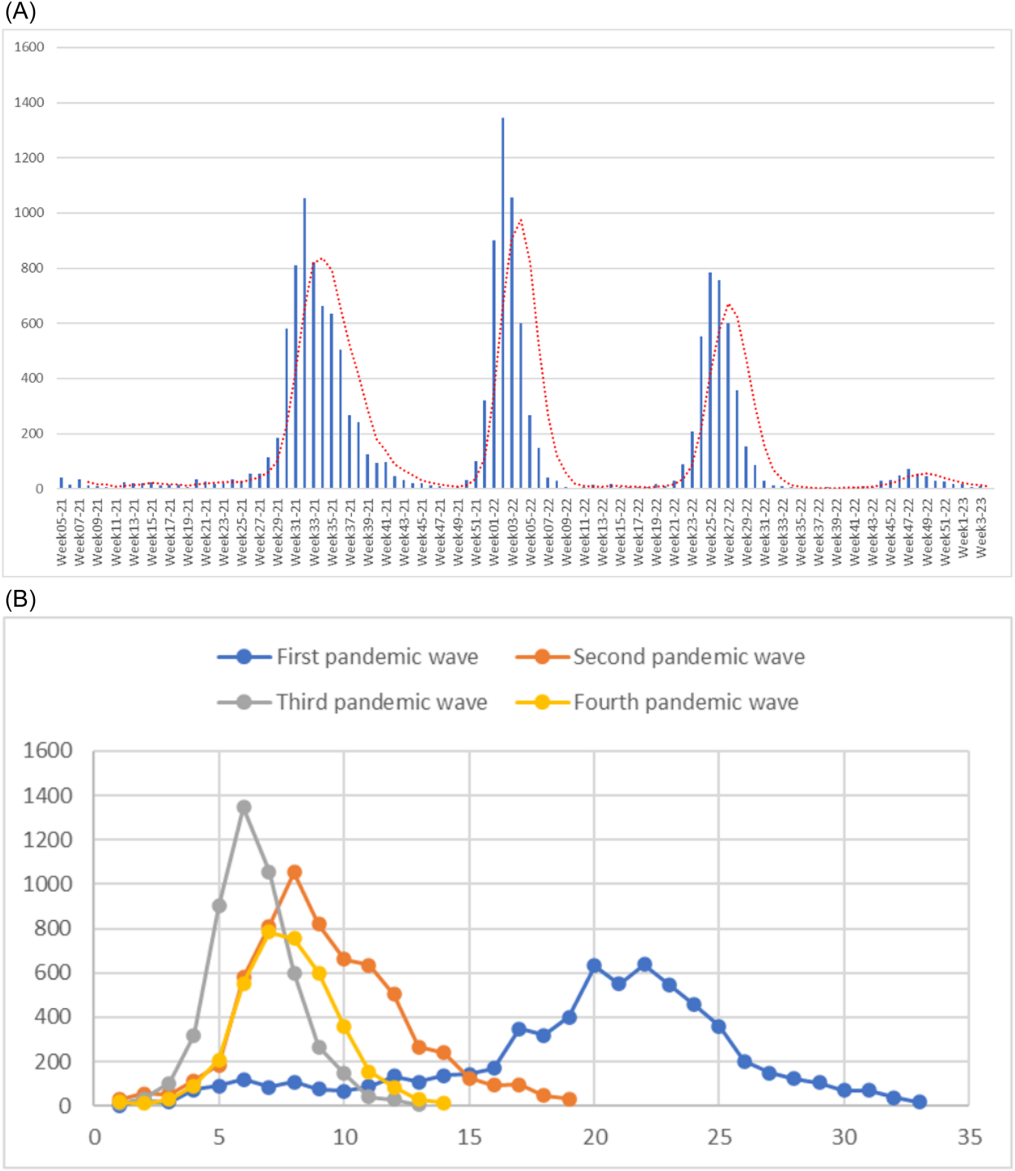
(A) The weekly evolution of SARS‐CoV‐2 positive cases diagnosed at the CVMIT—HMIMV using the real‐time RT‐PCR, in the period between March 2020 and January 2023: (B) Duration of COVID‐19 epidemic waves in our institutional database during March 2020 to January 2023 (weeks). These data are based on the number of SARS‐COV‐2 positive cases diagnosed by real‐time RT‐PCR, showing four pandemic waves represented with different colors: blue, red, gray, and yellow for the first, second, third, and fourth waves respectively.

Figure 3A,B depict the weekly evolution of diagnosed cases at the CVMIT belonging to HMIMV and the duration of COVID‐19 epidemic waves respectively. The incidence of cases began to spike during the initial wave, spanning from Week 27–20 (June 2020), and subsequently declined until Week 06–21 (February 2021), with the highest number of cases, reaching a maximum of 637, being recorded during Week 48 (November 2020). The second wave of the hospital's epidemic, which began in Week 25–21 (June 2021) and resulted in a total of 1053 positive cases by week 32 (August 21), was primarily attributed to B.1.17 infections. In October 2021, there was a decrease in the aforementioned wave, and the AY.33 variant became prevalent in our CVMIT cohort. This variant was linked to a brief extension of the third wave, resulting in 1346 reported cases during Week 02–22. During the 13‐week Omicron wave of Covid‐19, spanning from Week 19–22 (May 2022) to Week 32–22 (August 2022), the total number of confirmed cases did not exceed 785. It is noteworthy that the vaccine rollout commenced in the last month of 2021.

**Figure 4:**
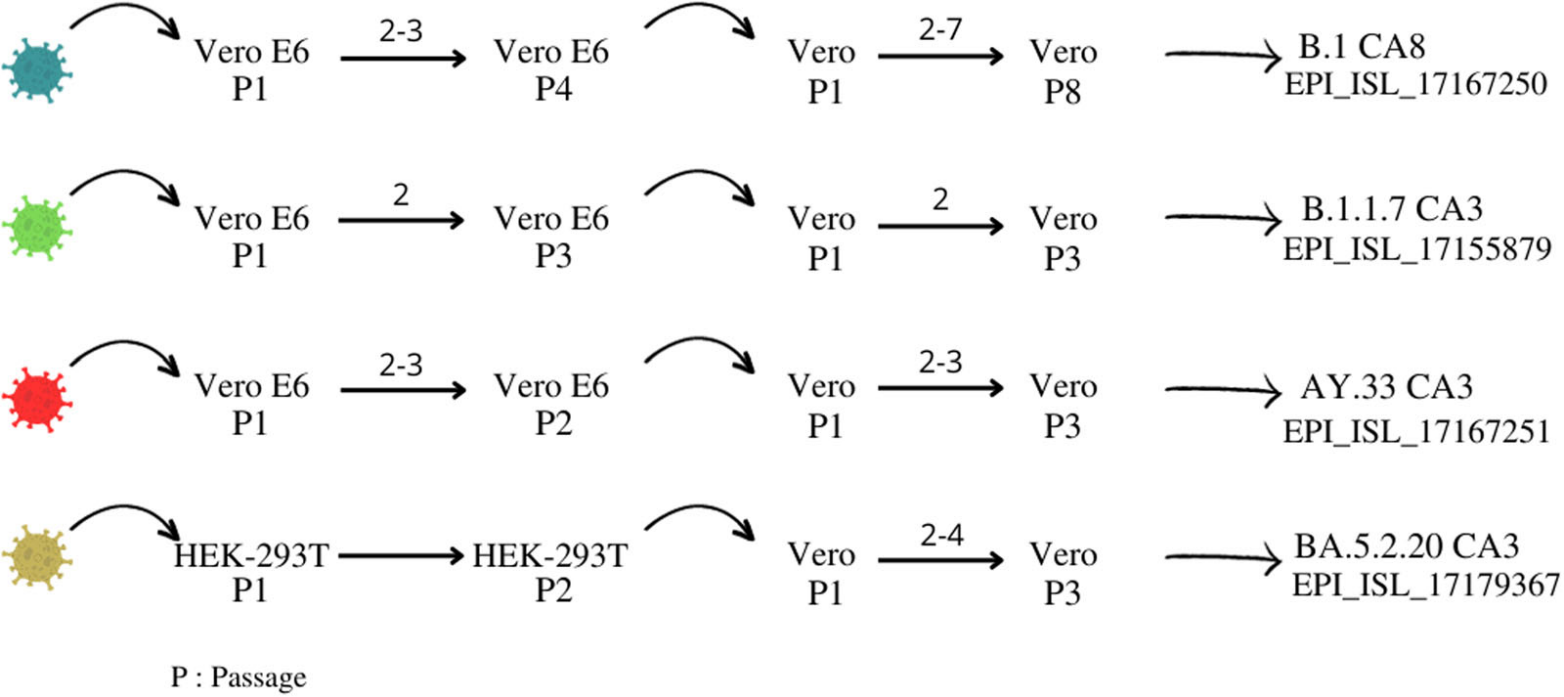
Cell‐passage numbering of the four SARS‐CoV‐2 variants isolated and used in our study, with the cell line used for isolation and virus culture of each strain. Each color represents one of the strains used in our study: blue, green, red, and brown beige represent the Wuhan D614G, Alpha, Delta, and Omicron variants, respectively.

Figure 4 and Table 1 show the cell‐passage numbering and amino acid (AA) changes in their Spike (S) proteins respectively. All strains conserved their parental AA changes in the S proteins, indicating the absence of genetic modifications during in vitro propagation.

### 3.2 Neutralization of B.1CA8, B.1.1.7CA3, AY.33CA3, and BA.5.2.20CA3 by immune sera derived from both control and vaccinated subjects

**Figure 5:**
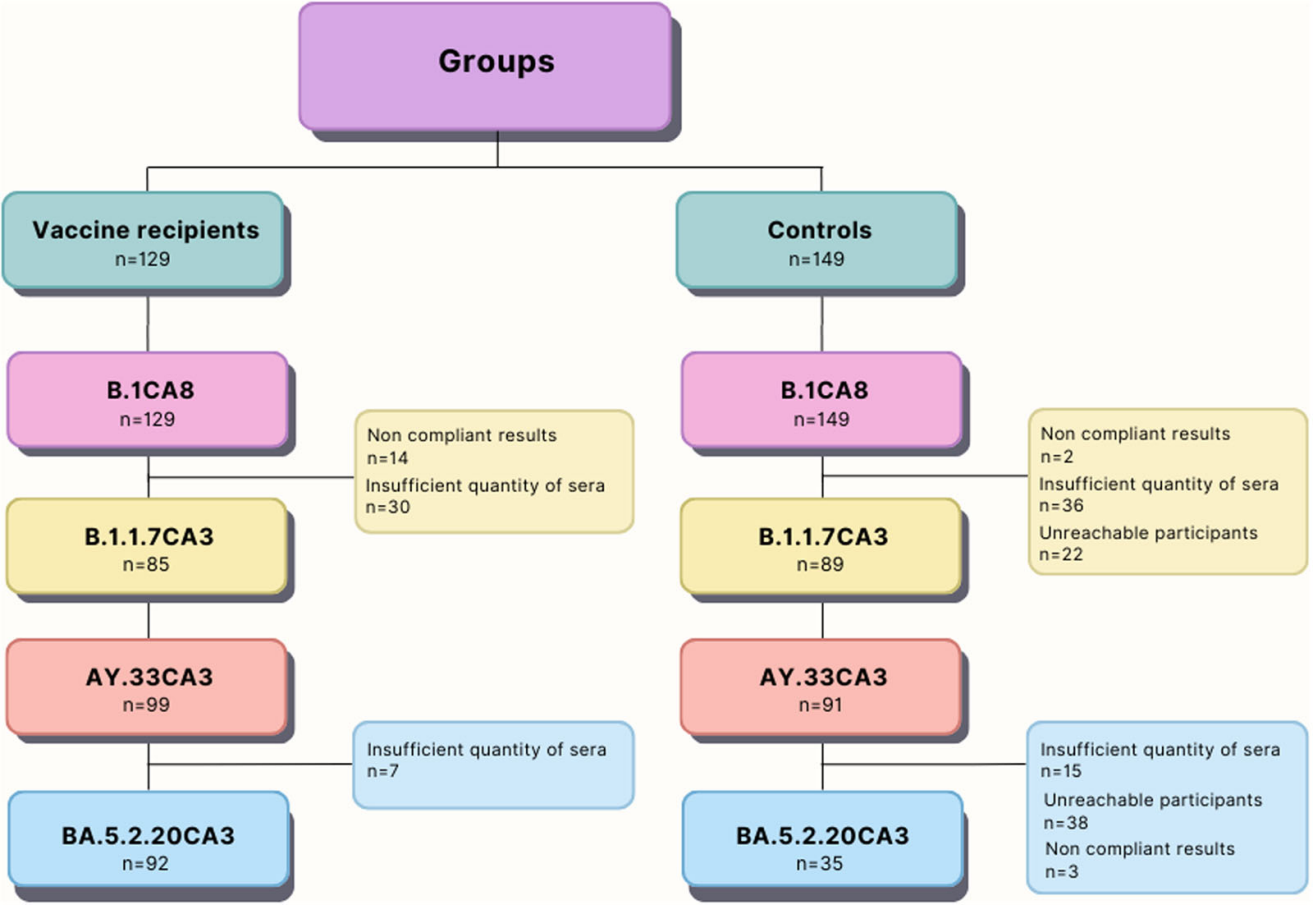
The flow diagram illustrates the progression of participant inclusion and exclusion at each stage of the analysis, delineated by vaccination status and against each SARS‐CoV‐2 strain used. Excluded participants at each stage are indicated, offering transparency regarding the reasons for participant exclusion.

First, the flow diagram (Figure 5) provides a visual representation of participant inclusion and exclusion throughout the analysis, segregated by vaccination status and against the four SAR‐CoV‐2 VOCs. It outlines the progression of vaccinees and controls through each analysis stage, indicating excluded participants at each step. This visualization aids in understanding participant selection and exclusion criteria, facilitating transparency and interpretation of study outcomes.

**Figure 6:**
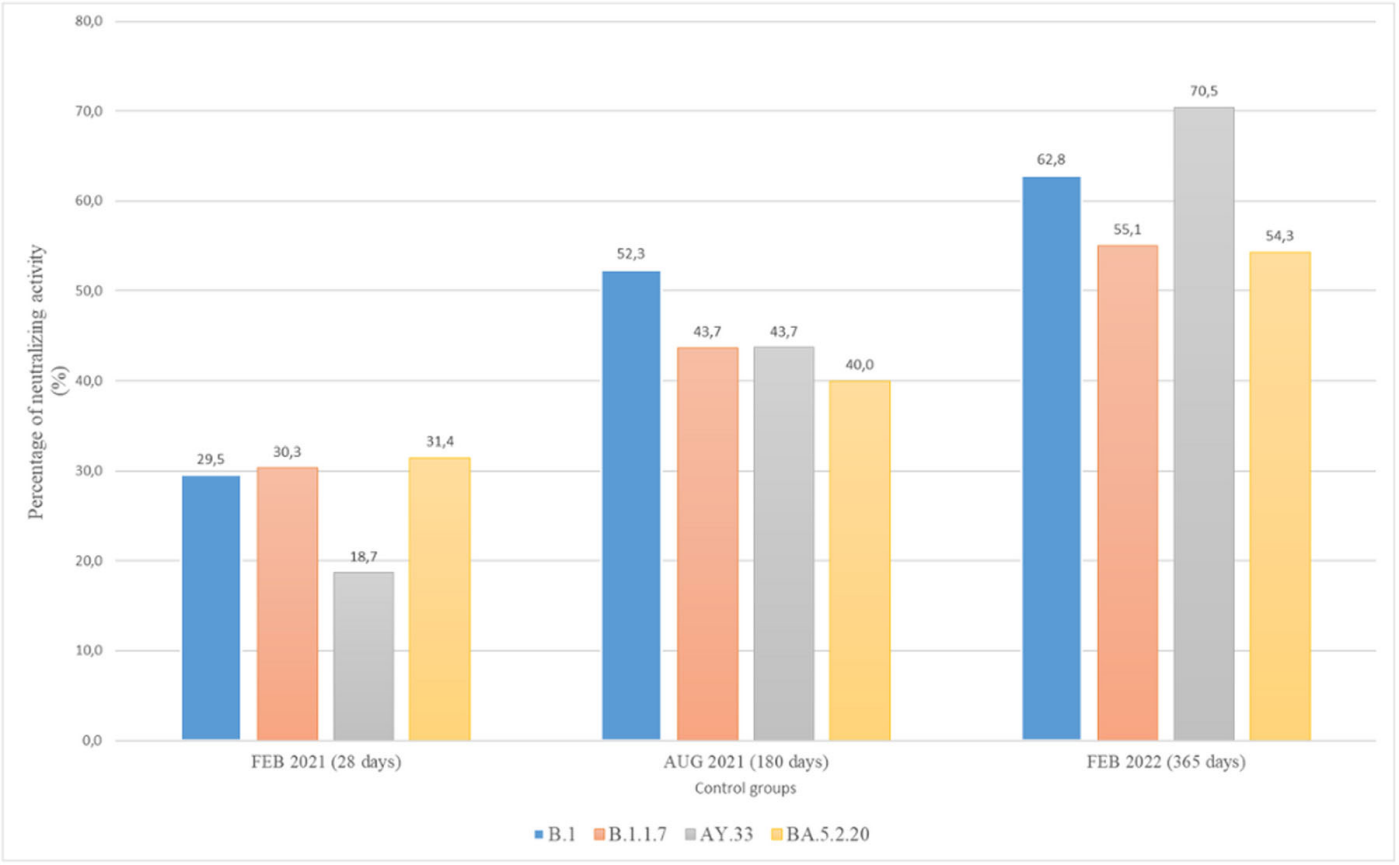
Neutralization activity rate (%) by immune sera from control groups against B.1, B.1.1.7, AY.33, and BA.5.2.20 at the time point of the study. Each color refers to a SARS‐CoV‐2 strain: blue, orange, gray, and yellow represent the Nabs of B.1, B.1.1.7, AY.33, and BA.5.2.20 respectively.

Of the participants, 86.6% were male and the median age was 35 years (IQR: 21–54). With regard to the humoral response towards all lineages, the proportion of seropositivity among the control group recruited during the study period exhibited a gradual increase. Specifically, the proportion increased from 29.5% to 62.8% against B.1CA8, from 30.3% to 55.1% against B.1.1.7CA3, from 18.7% to 70.5% against AY.33CA3, and from 31.4% to B54.3% for BA.5.2.20CA3. The data indicate that the group 1 cohort, who underwent screening, exhibited the lowest prevalence of S protein antibodies against AY.33CA3. Conversely, the highest prevalence was observed during the third round of testing in the winter term among group 3 in January 2022, as depicted in Figure 6.

**Figure 7:**
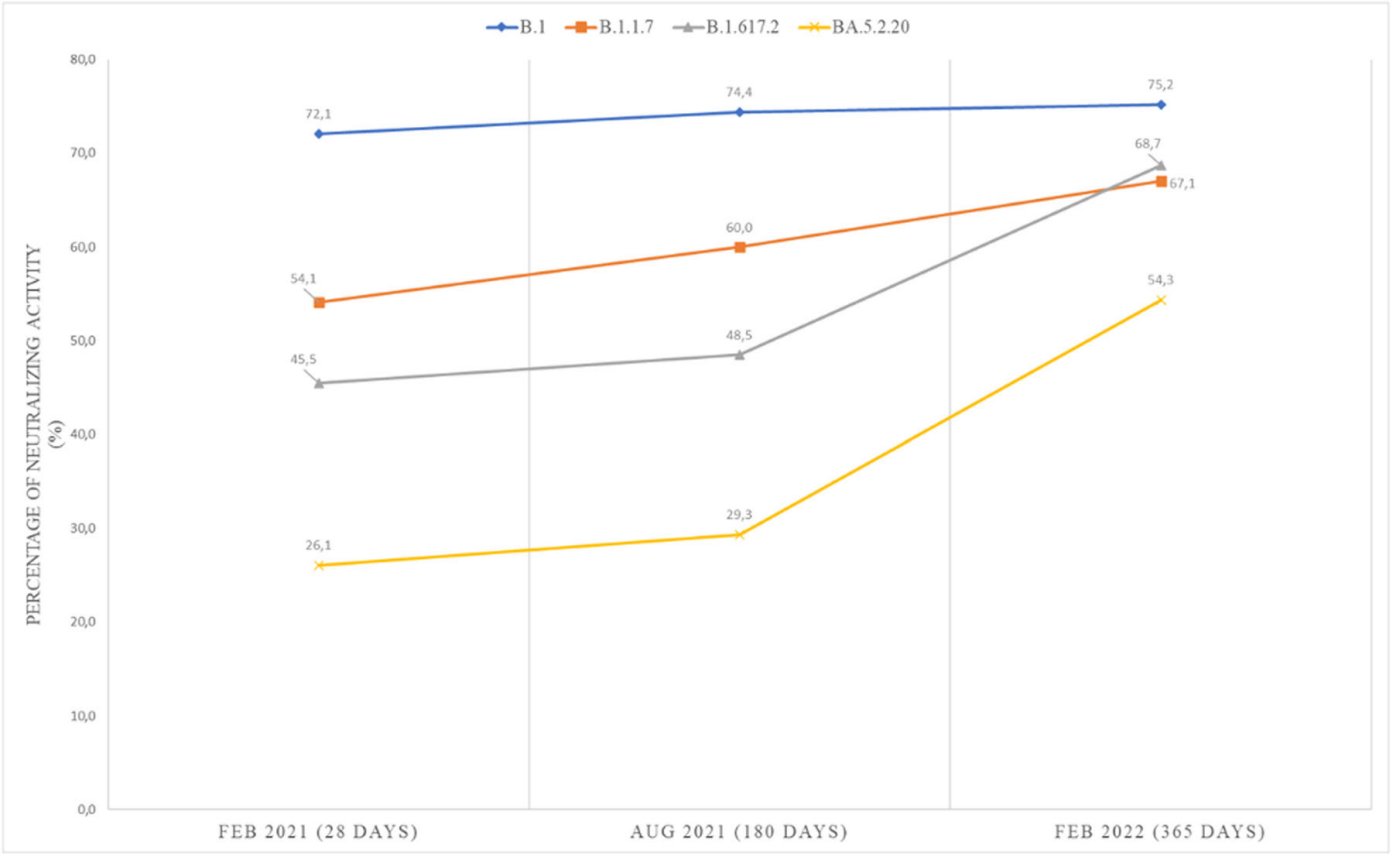
Neutralization activity of Nabs (%) of B.1, B.1.1.7, AY.33, and BA.5.2.20 at the time point of the study, by serum of two‐dose BBIBP‐CorV recipients, at the three key points. Each color refers to a SARS‐CoV‐2 strains: blue, orange, gray, and yellow represent the Nabs of B.1, B.1.1.7, AY.33, and BA.5.2.20, respectively.

Following the administration of the first dose of BBIBP‐CorV, no case of vaccine breakthrough infection (VBI) or infection among health workers were observed. The sera of vaccine recipients exhibited significant enrichment following vaccination, as evidenced by the antibody elicited at 28 days postvaccination (dpv). Ninety‐three vaccinated individuals (72%) showed titers ≥0.6 log10 against B.1, while 46 (54%) and 45 (45,5%) demonstrated such titers against B.1.1.7CA3 and AY.33CA3 respectively. Additionally, 24 (26%) sera displayed neutralizing antibodies against BA.5.2.20CA3. Meanwhile, NAbs activity of all the lineages was found to increase over time, with higher neutralizing activity against B.1.1.7CA3 (Alpha) (Figure 7).

Over 6 months, it was observed that the NAbs activity of all lineages increased against B.1CA8 (*n* = 51) and AY.33CA3 (*n* = 48). Furthermore, after 1‐year postvaccination, the number of participants with broad NAbs against AY.33CA3 (*n* = 68) and BA.5.2.20CA3 (*n* = 50) increased in response to the pandemic waves in Morocco. Conversely, there was an observed elevation of approximately 2%–3% in the neutralizing rate of vaccine recipients after 6 months, resulting in a heightened neutralizing potency against all lineages, as illustrated in Figure 6. During the third wave in Morocco, a total of five health workers, representing 3.85% of the sample, exhibited VBI. The median time from the second dose of BBIBP‐CorV until VBI was 156 days, with an interquartile range of 100–190 days.

### 3.3 Vaccine‐induced sera cross‐neutralizing activity against the VOCs

**Figure 8:**
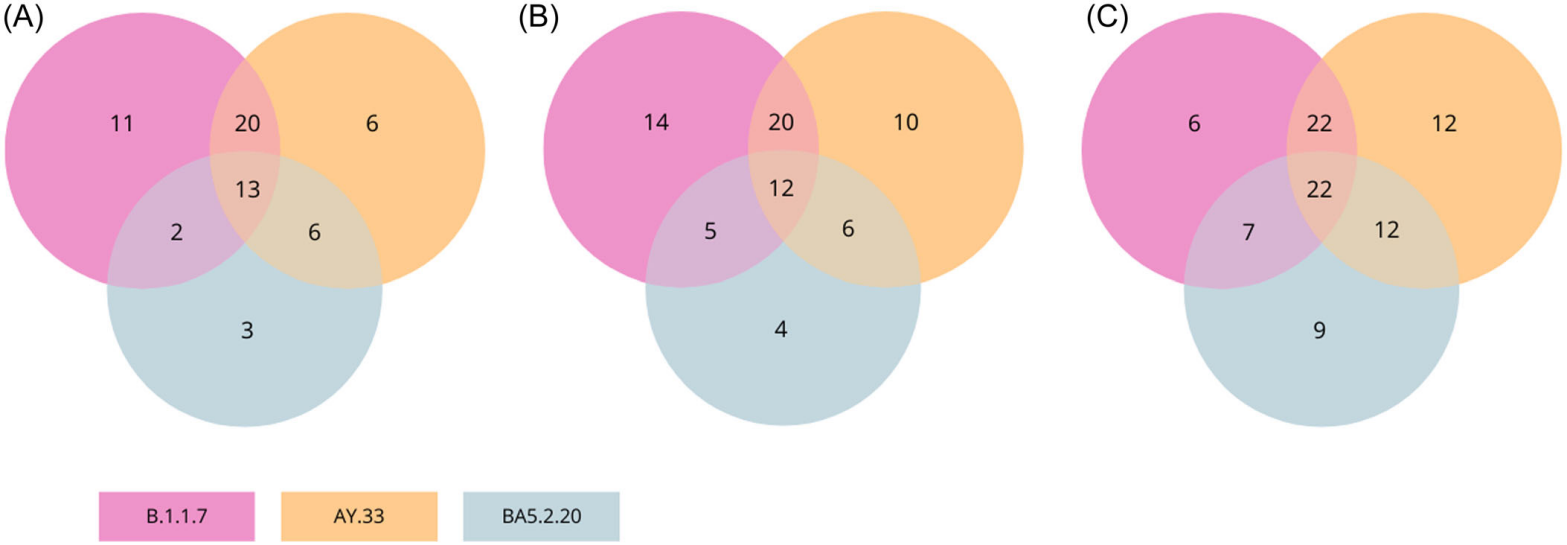
Cross‐neutralizing activity of NAbs with the three variants of concern (Alpha, Delta, and Omicron variant). Each Venn Diagram represents the cross‐reactivity within the vaccinated group at the three time‐point: 28 (A), 180 (B), and 365 dpv (C). Each color represents a positive neutralizing activity against the variant. Purple, orange, and blue refer to the activity against B.1.1.7, AY.33, and BA5.2.20, respectively.

The Venn diagrams depicted in Figure 8 serve to illustrate the relationships between vaccine‐induced sera that possess neutralizing antibodies capable of cross‐neutralizing variants as well as those that do not, with varying degrees of efficacy. Each sphere denotes distinct sections that exhibit either exclusive or intersecting characteristics, indicating the number of participants whose NAbs may have shared antigenic properties against VOCs. Upon analyzing the vaccine‐induced sera, 13 participants showed the ability to broadly neutralize the four lineages of the SARS‐CoV‐2 virus (out of the 93 participants). The findings were quite intriguing. It showed that four and seven individuals exhibited NAbs over a span of 1 year and 6 months postvaccination respectively. In August 2021 (6 months pv), five newly diagnosed sera exhibited broad neutralizing antibodies. Among these cases, four individuals maintained their strong neutralizing capacity throughout the entire 6‐month study. Moving forward to February 2022, an increase in the overall proportion of sera containing broad Nabs was observed. This increase amounted to 22 samples, with 14 of the sera belonging to health workers who had received two doses of the inactivated virus vaccine BBIBP‐CorV. These individuals had presented with probable VBI during the third wave of COVID‐19 in Morocco, as depicted in Figure 3A.

### 3.4 Correlation between the neutralizing activity of vaccinated and nonvaccinated groups

The statistical test revealed a positive correlation between the vaccinated and nonvaccinated groups, indicating that the vaccine elicits a higher neutralizing activity and affords superior protection against B.1CA8 and B.1.1.7CA3 over 1 year, in comparison to natural infection. In March 2021, a significant reduction in neutralization titer for AY.33CA3 was observed among the control group when compared to vaccine recipients (*p* < 0.0001). However, in January 2022, the MNT results for AY.33CA3 and BA.5.2.20CA3 didn't show any statistically significant difference (*p* > 0.5) (Table 2).

**Table 2:** The statistical resulting table depicts frequencies and percentages, comparing individuals receiving the two‐dose BBIP‐CorV vaccine to a control group.

		Vaccination status				
		−		+		*p* Value
		Frequency (*n*)	Percentage (%)	Frequency (*n*)	Percentage (%)	
28 dpv	B.1CA8	44	29.5	93	72.1	0.000
	B.1.1.7CA3	27	30.3	46	54.1	0.002
	AY.33CA3	17	18.7	45	45.5	0.000
	BA5.2.20CA3	11	31.4	24	26.1	0.657
180 dpv	B.1CA8	78	52.3	96	74.4	0.000
	B.1.1.7CA3	38	43.7	51	60	0.034
	AY.33CA3	38	43.7	48	48.5	0.557
	BA5.2.20CA3	14	40	27	29.3	0.291
365 dpv	B.1CA8	81	62.8	97	75.2	0.043
	B.1.1.7CA3	49	55.1	57	67.1	0.121
	AY.33CA3	62	70.5	68	68.7	0.874
	BA5.2.20CA3	19	54.3	50	54.3	1.000

*Note*: The *p* Value (in red) less than 0.05 didn't show any statistically significant difference.

The additional statistical analysis of the attributable fraction (Table 3) showed that the neutralizing activity against the Wuhan D614G and Alpha variant was due to the vaccination, at the three time‐points. Indeed, our findings showed that no significant difference between the vaccinated and control groups for delta and Omicron variants was noticed, at the three time‐points. As a result, the neutralizing activity was due to vaccination and field infection for Delta and Omicron SARS‐CoV‐2 VOCs.

**Table 3:** The statistical resulting table of the attributable fraction, comparing the vaccinated group to the controls.

		OR	AF
28 dpv	B.1CA8	6.12	0.836
	B.1.1.7CA3	2.71	0.631
	AY.33CA3	3.63	0.724
	BA5.2.20CA3	1.61	0.378
180 dpv	B.1CA8	2.65	0.623
	B.1.1.7CA3	1.93	0.482
	AY.33CA3	1.21	0.173
	BA5.2.20CA3	1.286	0.222
365 dpv	B.1CA8	1.8	0.444
	B.1.1.7CA3	1.66	0.398
	AY.33CA3	1.087	0.080
	BA5.2.20CA3	3.125	0.680

## 4 DISCUSSION

Our study aimed to analyze the immunological reaction elicited by the Sinopharm BBIB‐CorV vaccine, in response to SARS‐CoV‐2. Furthermore, we aimed to evaluate the capacity of sera samples derived from immunized individuals to neutralize SARS‐CoV‐2 variants of concern (Alpha, Delta, and Omicron).

The trajectory of COVID‐19 cases within our institution from the initial outbreak in March 2020 aligns with the pattern documented by the national governing bodies. The Moroccan Ministry of Health has released data, indicating that from March 2, 2020, to September 21, 2020, the proportion of asymptomatic cases upon admission was 74.9%, while the mild cases were 14.1%, moderate cases accounted for 9.6%, severe cases accounted for 1%, and critical cases accounted for 0.4%.^8^ Furthermore, our findings indicate that compared to the first wave, the second and the third waves exhibited greater severity and affected a larger population, which spanned 20 weeks. After December 2021, the fourth wave of the Omicron variant disseminated expeditiously, affecting a substantial populace, as reported by Amicone et al.^15^ In our investigation, it is noteworthy that the duration between the waves and the sera collection was 4 weeks subsequent to the first wave in control group 1, during the second wave for group 2, and the Omicron wave for group 3. During the study period, a rise in antibody seroprevalence was observed, among the control group may be accounted for by this phenomenon.

Cross‐neutralization assays were conducted in vitro, using sera from control and vaccine groups. The assays were performed compared to the first wave against four distinct isolates, which were collected at different times during the pandemic waves, compared to the first wave. The propagation of all strains was conducted in Vero cell lines. The identification of potential mutations within the viral population during cellular cultivation was ascertained through a comprehensive analysis of complete genomic sequences of both the parental and the cell‐passaged strains used in our study. The genomic integrity of SARS‐CoV‐2 strains in the virus spike glycoproteins surrounding the multi‐basic cleavage site remained unaffected, which is a noteworthy observation. The findings presented in this study are inconsistent with prior research that has shown the occurrence of genetic alterations during in vitro cultivation.^16^ The observed dissimilarities could potentially be attributed to variations in cultural methodologies. Such as variances in cell lines, variants, inoculated viral MOI during propagation, and the number of passage stocks. Our objective is to establish the neutralization capacity of antibodies produced postadministration of a two‐dose vaccine. Therefore, we will not be disclosing the complete intra‐sample genetic diversity present within the cultured stocks at this time, as this information is currently being prepared for another publication. The investigation of the SARS‐CoV‐2 spike protein across all strains revealed that viruses cultured in Vero cells retained the genuine features of their natural counterparts. This makes them appropriate for precise evaluation of the immunogenicity of the two‐dose BBIBP‐CorV vaccine.

The studies conducted by Xia et al.^17^ and Zhu et al.^18^ revealed that the MNT performed with the B.1 isolate which possessed amino acid variability at positions 78 and 614, resulted in antigenic matching of Nabs in 73% of sera collected, at 28 dpv with the vaccine strain included in the BBIBP‐CorV vaccine. The results of our study are comparable to those observed in the clinical trial involving a two‐dose regimen of 4 μg of BBIBP‐CorV vaccine on Day 28.

This percentage (73%) of vaccine convergence is found approximatively similar to that reported by the following studies. The findings of the phase III clinical trial conducted in Abu Dhabi indicate that the vaccine exhibits an efficacy rate of 86% in preventing symptomatic cases, and a 100% efficacy rate in preventing severe cases.^19^ A study was conducted in Chile from February 2 to May 1, 2021, involving 10.2 million participants (16 years or older). The study found that the Sinopharm inactivated vaccine exhibited an effectiveness rate of 65.9% against COVID‐19 infection, 87.5% against hospitalization, and 90.3% against death.^20^ A study conducted in Morocco from February 1 to June 30, 2021, evaluated the efficacy of the BBIBP‐CorV vaccine in a real‐world work setting. The result indicated that the Sinopharm vaccine demonstrated a comparable effectiveness of 89% across all age groups ranging from 18 to 99 years.^5^ Moreover, a Moroccan national study was conducted to examine the humoral immune response and the seroprevalence of Nabs among 177 individuals who received two doses of ChAdOx1 nCoV‐19 (AstraZeneca). The sera were obtained during April and May 2021, and exhibited a significant level of neutralizing activity (84%) against a SARS‐CoV‐2 strain as reported in Souiri et al.^21^

The findings indicate a decline in the immune response of individuals who received the vaccine, as observed during the 1‐year follow‐up period. The decrease in question is an inherent aspect of the Nabs and is subject to a multitude of factors. Nevertheless, a reduction in the levels of antibodies does not inevitably imply that an individual is entirely immune to the virus. Multiple research studies have examined the persistence of immune responses in individuals who have received the BBIBP‐CorV vaccine in relation with SARS‐CoV‐2 variants. These studies have revealed a reduction in neutralizing activity. According to a study conducted in China, there was a decrease of around 50% in antibody titers 6 months after the second dose.^22^ A comparable outcome was observed in a study conducted in the United Arab Emirates, albeit with a lesser degree of reduction in those who had been administered a third booster shot of the BBIBP‐CorV vaccine.^23^

Notwithstanding the observed decline in neutralizing activity, it is important to notice that a considerable of vaccinated individuals retained a moderate level of immunity against the alpha and delta variants, as per our findings. The phenomenon of immune escape can be attributed to several factors, of which are mutations in the viral spike protein. These mutations can lead to structural changes in the protein, which is the primary target of immunity response. As a result, the ability of T cells and neutralizing antibodies to detect and react to SARS‐CoV‐2 may be compromised. In addition, it should be noted that mutations occurring beyond the spike protein may have an impact on the sites recognized by neutralizing antibodies, thereby potentially contributing to immune evasion.^24^, ^25^

The Alpha B.1.1.7 variant has been linked to a decrease in the neutralization capacity of monoclonal and natural antibodies due to the presence of mutations N501Y and E484K.^26^, ^27^ Certain mutations in the Spike protein of the Delta variant, such as P681R and D950N, have been found to result in heightened viral replication and potential immune evasion.^28^ Several research studies have demonstrated the ability to enhance virulence, transmissibility, and immune escape through the identification of mutations. According to a study working on the impact of Omicron variant mutations, the aforementioned variant, which was first reported in South Africa, has more than 30 mutations in the spike protein, with 10 of them being present in the receptor‐binding domain (RBD). Mutations E484A, G446S, and S477N, are common mutations with other VOCs, such as alpha, beta, and gamma. These mutations have also been linked to immune evasion.^29^, ^30^

Ongoing research is being conducted to examine the neutralizing cross‐reactivity of vaccines against VOCs. Our findings indicate that 13 sera have the ability of cross‐neutralizing the B1CA8 and the three VOCs (Alpha, Delta, and Omicron variants) on 28 dpv. Some of the sera maintained their neutralizing activity for up to a year, while others in the vaccine group developed the ability to broadly neutralize over time. According to a recent study, it has been shown that sera from individuals vaccinated with the BBIBP‐CorV vaccine exhibit neutralizing activity against the Alpha, Beta, and Delta variants, although at lower levels compared to the wild‐type virus.^9^ Vaccines have the potential to generate cross‐neutralizing antibodies against various SARS‐CoV‐2 variants. However, the neutralization potency of these antibodies may be diminished when it comes to variants of concern, including but not limited to Alpha, Delta, and Omicron. The findings indicate that the BBIBP‐CorV vaccine elicits immunity against SARS‐CoV‐2 and has the potential to generate cross‐neutralizing antibodies against the VOCs. However, the extent of cross‐reactivity and efficacy may be influenced by various factors.

The findings of our study indicate that the vaccine‐induced neutralizing activity is superior in protecting against B.1CA8 and B.1.1.7, compared to the neutralizing activity resulting from field infection (control groups), over 1 year. Moreover, the neutralization titers for AY.33 were significantly lower in the control groups in March 2021, as compared to the vaccine recipients. The findings demonstrate the efficacy of the BBIBP‐CorV vaccine administered in two doses in conferring increased immunity against the B.1 D614G and B.1.1.7 variants. Several studies have demonstrated the efficacy of vaccines in mitigating the impact of SARS‐CoV‐2 variants of concern. Notably, one study observed comparable outcomes to those of the wild‐type strain, albeit with reduced neutralizing activity against the Beta and Gamma variants.^31^ A recent investigation revealed that the serum samples collected from individuals who received the vaccine exhibited diminished neutralizing efficacy against the P.1 variant in contrast to the initial SARS‐CoV‐2 strain.^32^ Another study conducted in Argentina aimed to assess the humoral response over time in healthcare workers who received the BBIBP‐CorV vaccine. The study reported a decrease in antibody levels over time among Sinopharm recipients.^33^

In Morocco, few studies have been conducted on anti‐SARS‐CoV‐2 IgG levels using Immuno‐serological tests. Two studies worked on vaccinated participants investigating the humoral response. The first aims to determine anti‐SARS‐CoV‐2 anti‐S IgG levels among 82 healthcare care workers 5 months after the second vaccination dose (ChAdOx1 nCoV‐19 AstraZeneca or BBIBP‐CorV Sinopharm vaccine). It provided information on the longevity of anti‐SARS‐CoV‐2 IgG antibodies in individuals. 65,85% of participants had anti‐SARS‐CoV‐2 IgG antibodies, including 24 (44.44% who were vaccinated with Sinopharm. This study also reported that the antibody positivity rate was higher for positive men vaccinated with Sinopharm compared to the positive women group.^34^

The second research is on a total of 46 healthcare workers, to evaluate the humoral response after a third dose of mRNA BNT162b2 Pfizer or BBIBP‐CorV Sinopharm vaccine who were fully vaccinated with AstraZeneca (Two‐dose). It shows that the BNT162b2 Pfizer vaccine elicits a significantly higher antibody level compared to the BBIBP‐CorV vaccine.^35^

Our study has some limitations that should be considered. The impact of age, gender, and comorbidities on neutralizing activity was not investigated. Additionally, examining the correlation between the kinetics of antibody responses and neutralizing titers could provide valuable insights. However, this study is the first study in North Africa to access the immunogenicity of the two‐dose BBIBP‐CorV Sinopharm vaccine of health workers using a gold standard method. In Morocco, several studies assessed the effectiveness or immunogenicity of the SARS‐CoV‐2 vaccine using serological techniques (ELISA) and epidemiological analysis. Few of them were conducted on the BBIBP‐CorV Sinopharm vaccine. This study also provides information on the duration and persistence of neutralizing antibodies against three SARS‐CoV‐2 VOCs (Alpha, Delta, and Omicron) for up to 1 year.

## 5 CONCLUSIONS

The immunogenic response of a two‐dose immunization by the inactivated SARS‐CoV‐2 Sinopharm vaccine achieved higher neutralization activity against SARS‐CoV‐2 B.1 and B.1.1.7 (Alpha), compared to field infection (control groups), over 1 year. However, it is comparatively lower against the newly emerging VOCs of SARS‐CoV‐2 (Delta and Omicron) for the vaccinated group. The findings suggest that the immunogenicity of the BBIBP‐CorV (Sinopharm) COVID‐19 vaccine, administered in two doses, is relatively low, in terms of neutralizing the SARS‐CoV‐2 VOCs. As a result, it is recommended that additional boost vaccinations be considered.

## AUTHOR CONTRIBUTIONS

**Maha Ouarab**: Conceptualization; data curation; formal analysis; methodology; writing—original draft. **Elarbi Bouaiti**: Formal analysis. **Zineb Rhazzar**: Investigation. **Hicham El Annaz**: Resources. **Safae el kochri**: Investigation. **Mouhssine Hemlali**: Investigation. **Hamza Ghammaz**: Formal analysis; software. **Omar Nyabi**: Investigation. **Karim el Bakkouri**: Writing—review & editing. **Nadia Touil**: Conceptualization; methodology; project administration; resources; supervision; validation; visualization; writing—review & editing. **Mostafa Elouennass**: Investigation. **Lamiae Belayachi**: Investigation. **Jean Luc Gala**: Investigation. **Khalid Ennibi**: Investigation; resources; writing—review & editing. **Elmostafa El Fahime**: Conceptualization; funding acquisition; project administration; supervision; validation; visualization; writing—review & editing.

## ETHICS STATEMENT

The informed consent process was conducted in writing and was duly authorized and approved by the scientific committee of the military hospital and the ethical committee (CEFCZ/AB/30/01/2021 folder “PR _RFG_2021”).

## Data Availability

Data is contained within the article.
